# Fast Feedback in Active Sensing: Touch-Induced Changes to Whisker-Object Interaction

**DOI:** 10.1371/journal.pone.0044272

**Published:** 2012-09-18

**Authors:** Dudi Deutsch, Maciej Pietr, Per Magne Knutsen, Ehud Ahissar, Elad Schneidman

**Affiliations:** 1 Department of Neurobiology, The Weizmann Institute of Science, Rehovot, Israel; 2 Departments of Physics, University of California at San Diego, La Jolla, California, United States of America; University of New South Wales, Australia

## Abstract

Whisking mediated touch is an active sense whereby whisker movements are modulated by sensory input and behavioral context. Here we studied the effects of touching an object on whisking in head-fixed rats. Simultaneous movements of whiskers C1, C2, and D1 were tracked bilaterally and their movements compared. During free-air whisking, whisker protractions were typically characterized by a single acceleration-deceleration event, whisking amplitude and velocity were correlated, and whisk duration correlated with neither amplitude nor velocity. Upon contact with an object, a second acceleration-deceleration event occurred in about 25% of whisk cycles, involving both contacting (C2) and non-contacting (C1, D1) whiskers ipsilateral to the object. In these cases, the rostral whisker (C2) remained in contact with the object throughout the double-peak phase, which effectively prolonged the duration of C2 contact. These “touch-induced pumps” (TIPs) were detected, on average, 17.9 ms after contact. On a slower time scale, starting at the cycle following first touch, contralateral amplitude increased while ipsilateral amplitude decreased. Our results demonstrate that sensory-induced motor modulations occur at various timescales, and directly affect object palpation.

## Introduction

Rats explore their proximal environment through periodic and non-periodic protractions of their mystacial vibrissae (whiskers). Laboratory rats can be trained to use their whiskers in a wide range of tasks [1], including texture discrimination [2], [3], distance and width judgment [4], [5], object localization [6], [7], [8], [9], and shape discrimination [10].

When a stationary rat whisks unobstructed, its whisker movements are generally highly periodic and bilaterally symmetric [11], [12]. Periodicity in the motor output persists during sensory nerve inactivation [13], as well as following cortical ablation [13], [14]. This suggests that a default periodic state of whisking is generated by a sub-cortical central pattern generator (CPG), rather than resulting from cycle-to-cycle feedback control. However, periodicity and symmetry often break down during head movements [15] or contact [16], [17], which suggests that both efferent and afferent signals can induce deviations from periodic whisking.

On-going control of whisking would permit adaptive sensory sampling in both time and space. Such adaptive control could rely on various sensory-motor loops, and may involve a CPG. In the rodent vibrissal system, there are multiple such pathways that form closed-loops between the primary afferents and motoneurons [1], [18], [19], [20]. Evidence collected so far suggests that different variables of active touch are controlled by different loops [21], [22], [23]. Different loops are characterized by different loop delays, determined by their connection schemes and strengths (e.g., [24]).

In behaving rats, whisking is modulated at various timescales, both shorter and longer than that of a single whisking cycle (“whisk” duration is typically 50–250 ms). Within-cycle modulations in which the whisker velocity changes its sign have been described as inducing “pumps.” “Single pump” or “delayed pump” have denoted whisks that had one peak in the angle trajectory. “Double pump” have denoted whisks that had multiple peaks [2], [25], [26]. Both rapid and slow modulations can result from top-down control of the whisking circuits and from ex-afferent or re-afferent sensory feedback [1],[27]. Here, we characterized active whisking patterns, during free-air whisking and whisking against an object, of awake, head-fixed rats using high-speed videography to track individual whiskers with high precision in time and space. We report two types of ex-afferent modulations: a within-cycle ipsilateral modulation and across-cycles bilateral modulation.

## Materials and Methods

### Animals

The whisking patterns of male albino Wistar rats (N = 7; 180–230 g) were measured. The rats were housed individually post-operatively on a normal 12 h light/dark cycle. Water was available *ad libitum*, except during experimental sessions when no water or other liquid was given. All whiskers were trimmed, except for three whiskers (C1, C2, and D1) on each side of the snout. This configuration was chosen to allow comparison both within a row and within a column, while having only a single rostral whisker. Whiskers were clipped close (∼1 mm) to the skin during brief (5–10 min) isoflurane anesthesia, and were re-trimmed two to three times a week at least 2 hours before an experiment.

### Ethics statement

Animal maintenance and all experimental procedures were conducted in accordance with the guidelines of the National Institutes of Health (USA) and The Weizmann Institute of Science.

### Surgery

Rats were fitted with head posts for head fixation, as previously described [28]. Briefly, animals were anesthetized with ketamine (70 mg/kg, i.m.) and xylazine (10 mg/kg, i.m.). The skull was exposed, and six miniature stainless steel screws (MX-0080-02B; Small Parts; Miami Lakes, FL) were drilled into the bone. Two larger screws, used as head-posts, were glued to the bone with cyanoacrylate glue, and all exposed bone and screws capped with a layer of acrylic cement (Jet Acrylic; Lang Dental Mfg.; Wheeling, IL). Post-operatively, rats were treated with antibiotics (Penicillin and Streptomycine; ‘Pen & Strep’, Norbrook Inc; 2 ml/kg, s.c.), analgesics (Carprofen; 5 mg/kg, s.c.), and food and water *ad libitum* for a minimum of 6 days.

### Head fixation

Rats were allowed to recover from surgery for at least 6 days prior to head fixation, after which behavioral training commenced. In each training session, rats were immobilized by placing them inside a nylon bag and a plastic tube (6 cm in diameter). During the first two or three days of behavioral training, the rats were restrained in this manner while their heads were held in place by hand for up to 15 min. The duration of immobilization was gradually increased, and at 4 to 5 days post-recovery, their heads were held in place by an articulating arm (NF60101; Noga Engineering; Shlomi, Israel) that was mechanically connected to the fixation tube, for increasingly longer periods of immobilization. The maximal time for head fixation in either the adaptation or experimental stages was 30 min. Head fixation was terminated earlier if rats showed signs of distress. During the adaptation stage, rats were trained only once a day. During the experimental stage, each rat had one or two experimental sessions a day, two or three times a week.

### Experimental apparatus

**Figure 1:**
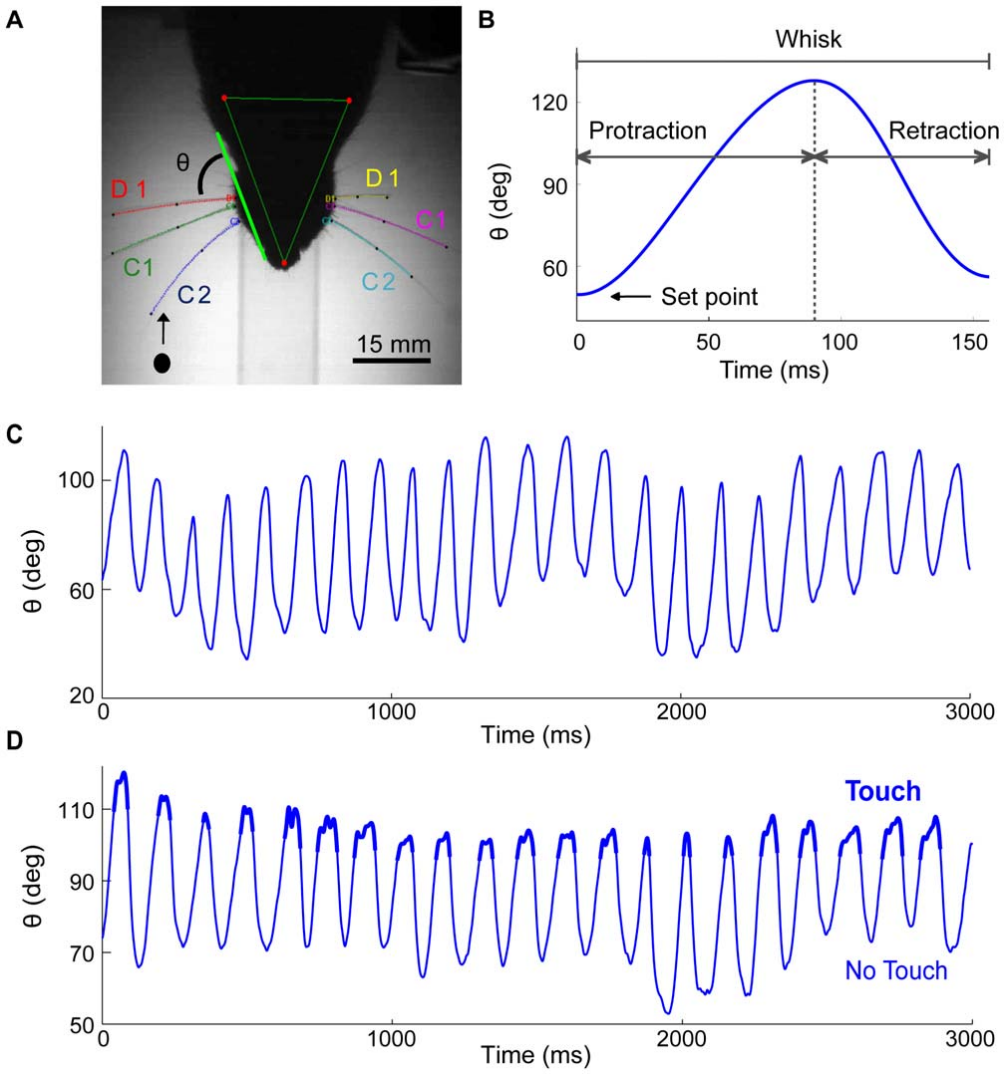
Acquisition and pre-processing of whisker trajectories in rats. **A.** Overhead view of a head-fixed rat with whiskers C2, C1, and D1. The instantaneous whisker angle, θ, was defined as the angle between the whisker base and the nose-eye line. The pole is marked schematically in its typical initial location, with an arrow indicating the direction of its motion into the whisking field of the rat. **B.** The protraction and retraction phases of a single whisk are depicted in relation to whisker angle and protraction onset time. The angle of the whisker on the whisk's onset (set point) is indicated by an arrow. **C.** Example of a free-air whisking trajectory (filtered at 80 Hz). **D.** Example of a whisking trajectory against an object (filtered at 80 Hz). Whisker-pole contact is indicated by bold. In this example, the rat made 21 successive whisks with touch.

All experiments were performed in a dark, sound-isolated chamber. Whisker movements were recorded at 480×640 pixels resolution using a high-speed video camera (500–1000 frames/s; MotionScope PCI; Redlake; San Diego, CA) placed ∼50 cm above the discrimination area. Head orientation was estimated by imaging the corneal reflections of two infrared (880 nm) light-emitting diode (LED) spotlights (F54845; Edmund Industrial Optics; Barrington, NJ), placed 10–15 cm above the animal (Fig. 1A; top view). An imaginary line between the nose and eye on each side of the rat served as a reference line for the whisking angle (θ; Fig. 1A). Bright-field imaging of the whiskers was accomplished by projecting infrared light (880 nm) with an array of 12×12 LEDs (StroboLED II; AOS technologies AG; Baden-Daettwill, Switzerland) from below the animal. Video acquisition was triggered manually, and high-speed video was buffered and streamed to disks at either 500 frames/s (N = 163 experimental sessions) or 1,000 frames/s (N = 92 experimental sessions).

### Experimental procedure

A total of 255 trials with an average duration of 8.3±3.2 s (mean ± standard deviation, SD) were acquired. In 106 “free-air trials”, all whisking was made in free-air with no obstacles presented. In 149 “contact trials”, a vertical metal pole (2.8 mm in diameter), attached to a micron resolution linear actuator (Abiry; Tel Aviv, Israel), was moved into the whisking range on either the right or left side of the snout. In contact trials, video was acquired from 3.5±2.5 s prior to first contact, until 4.3±2.4 s after it. Onset of object movement was triggered manually when rats self-initiated a bout of rhythmic whisking. The object was then moved at an average velocity of 1.1 cm/s to a predetermined location inside the field of the moving whiskers. The position of the object was chosen such that whisker C2 would touch the object at a radial location of about 40% of the whisker length (∼30 mm), which is within the normal contact-range during exploratory whisking (the contra-lateral asymmetry reported by [16] in freely moving rats was most prominent for a subset of data where the wall was within 10 mm of one wall; see also [6]). Across all contact trials, the average whisker angle at touch was 87.9±10.7 deg (mean ± SD), and the distance between the whisker base and the contact point was 9.5–12.5 mm.

Typically, rats whisked continuously throughout contact trials, which allowed comparison of whisking behavior before and after the first touch. In most contact trials, the first touch occurred while the object was still moving. The acoustic noise induced by the motor was within the auditory range of the rats, and the effects of pole movement initiation on whisking were therefore tested (see Results). Since each trial was initiated manually when whisking was detected by the experimenter, the ratio of whisking periods (“bouts”) to non-whisking periods does not reflect the overall statistics of head-fixed rats. Free-air trials (N = 106) were used both for characterization of whisking in free air, and as a control for touch induced effects. Free-air and contact trials were randomly intermingled during each experimental session.

### Whisker tracking

**Figure 2:**
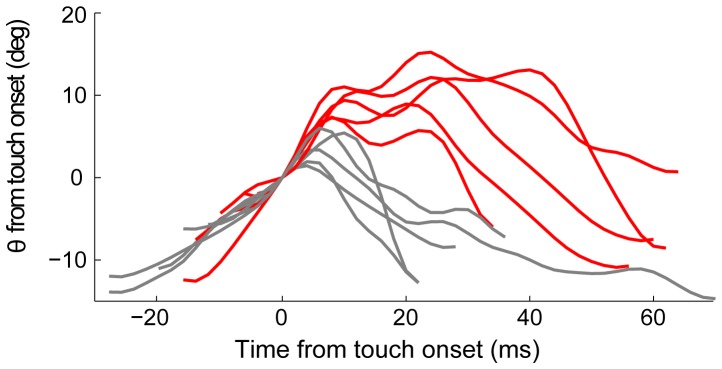
Whisking and head motion in freely moving rats. Examples of whisker trajectories (filtered at 80 Hz) from freely-moving rats performing a localization task [6], demonstrating the occurrence of whisks with a single peak (gray) and multiple peaks (red) after contact onset.

**Figure 3:**
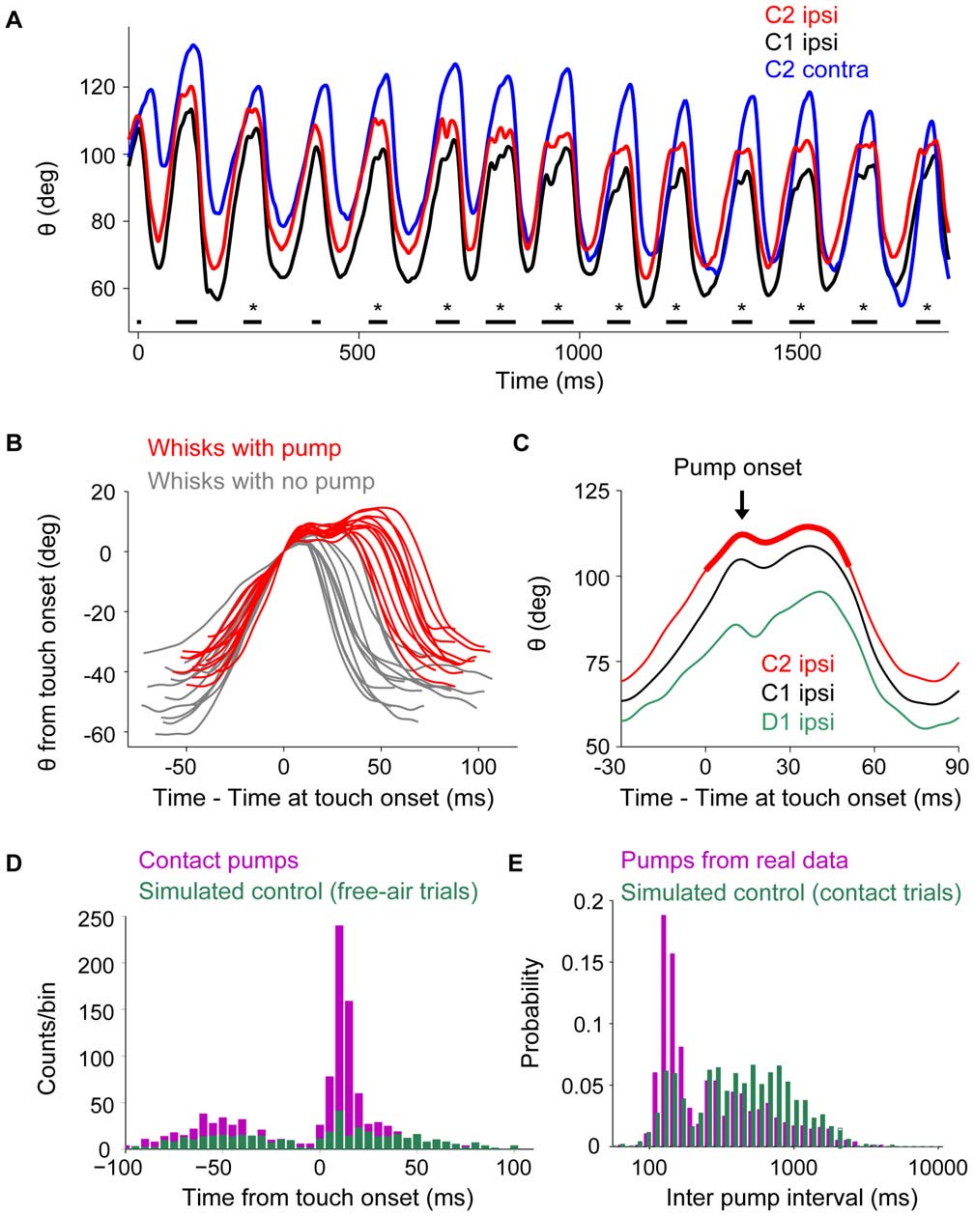
Fast, within-cycle touch-induced ipsilateral feedback. **A.** Angle trajectory from a contact trial, filtered at 80 Hz. Whisker C2 (red) touched the pole near the end of protraction. Touch events are indicated by black horizontal lines. TIPs (manually detected in this example) are indicated by asterisks. The first touch depicted was also the first touch in the trial. Data for ipsilateral C2 (red), ipsilateral C1 (black), and contralateral C2 (blue) whiskers. **B.** Angle trajectory of whisks with TIP (red) and without (grey), all taken from a single trial. Time and angle are presented with respect to their values at touch onset. **C.** Example of a touch-induced pump (TIP) that, after contact between whisker C2 and the pole, occurred simultaneously in the three untrimmed whiskers (C1, C2, D1). The C2 whisker was continuously in contact with the pole throughout the period marked touch (bold red line), so the whisker palpates against the pole without detaching from it. The TIP peak or onset (indicated by the arrow), the time of the first peak after touch, is followed by negative velocity. **D.** Time from touch onset (start of red bold line in C) to TIP onset (arrow in C) in contact trials (purple). The average latency of touch-induced pumps was 17.9 ms. The distribution of times between pseudo-touches (crossing of a threshold angle in free-air trials) and pump onset was normalized to have the same number of touches as in the contact data and is denoted in green. **E.** Inter-pump-interval (time between touch onsets in two neighboring whisks-with-TIP) with experimental (purple) and simulated (control; green) data. The control distribution is what is expected if the pumps had the same probability to occur, but were distributed randomly upon touch events. The peak around 140 ms is due to pumps that occurred in successive whisks (see Materials and Methods for detailed description on the controls used in Figs. 3D and 3E).

Whisker movements were tracked off-line using the *Matlab*-based *WhiskerTracker* image processing software (available at http://code.google.com/p/whiskertracker; [29]). Briefly, head location and orientation were determined from the corneal reflection of two overhead LEDs. The location of each whisker and its shape was estimated by fitting piecewise polynomials (splines) to the horizontal projections of whisker profiles in the video frames. Whisker angles were estimated with respect to the eye-nose line on the side of the tracked whisker (Fig. 1A), using the coefficients of a polynomial fit to the whisker portion proximal to the whisker base [29]. Since the rats were head fixed and most whiskers were trimmed, individual whiskers were easily identified, and their tracking was automatic in most cases. In particular, whisker roots along the arc (C1–D1) were easily separated when viewing from overhead (Fig. 1A). Whisking trajectories were low-pass filtered at 15 Hz prior to the automatic detection of individual whisks (Figs. 1B–D). A higher frequency filter (80 Hz) was used for the analysis of fast effects of contacts (Fig. 2; Fig. 3A–C), which ensured that trajectory peaks masked when filtered at 15 Hz would be detected. Whisks were classified automatically as cycles of protractions and retractions (see *Automatic detection of individual whisks*). Touch events (contacts and detachments) were identified manually by two independent observers. When a contact was detected by only one of the observers, or when duration of touch events deviated by more than 20 ms (which occurred with about 10% of the whisks being categorized manually), a third observer decided.

### Automatic detection of individual whisks

For detailed analysis, whisking trajectories were first low-pass filtered at 15 Hz. All minima and maxima in the trace were then parsed into a series of minimum to maximum traces (up traces), each followed by a maximum to minimum peak trace (down trace). Whisk amplitude was defined as the protraction amplitude, and whisk duration was defined as the combined duration of protraction and retraction. Set-point was the angle at the protraction onset (see Fig. 1B). For a single-pump whisk, the up trace is expected to be the protraction and the down trace the retraction phase. For double pump whisks, up and down traces are expected to represent also sub-components of individual whisks. Thus, threshold amplitude, R_th_, was determined such that only traces whose absolute amplitudes were larger than R_th_ were considered as up or down trace. A trace with smaller amplitude was merged with its successive trace. R_th_ was empirically determined as 2 deg, which was the lowest value that did not result in distinct modes in the resulting distribution. Whisk outliers were rejected based on either one of the following kinematic parameters: protraction amplitude, whisk duration, the ratio between protraction and retraction amplitudes, or the ratio between protraction and retraction durations. The distribution of whisk duration was approximately normal, so extreme values (nearly 2 standard deviation; 5% of the total) were excluded, which left whisk durations of 50–260 ms. The distribution of whisk amplitudes was not normal. R_th_ (see preceding section) was chosen as the minimal protraction amplitude. For amplitudes greater than 20 deg, their likelihood dropped linearly. The highest 1% of amplitudes was excluded, which left amplitudes of 2–70 deg. After these selections, remaining whisks were considered valid only if their duration ratios (protraction duration/retraction duration) and amplitude ratios (protraction amplitude/retraction amplitude), were between 1/θ and θ, where θ = 2.85. These additional criteria removed extreme events and were chosen to exclude the 5% tails of the whisks. The majority of excluded whisks was either outside whisking bouts, or at the start or end of bouts, and was frequently considered as irregular by human observers.

The trajectory from the onset of an up trace to the offset of the following down trace was defined as a single whisk. Overall, 10.6% of potential whisks were excluded based on their kinematics (amplitude, duration, and the ratio between amplitude or duration of protraction/retraction), which left 16,751 whisks over all whiskers in the trials with no pole (free air trials), and 33,688 whisks in contact trials.

### Definition and analysis of Touch Induced Pump (TIP)

Upon contact with an object, both contacting and non-contacting whiskers often decelerated, and then accelerated, resulting in additional inflection or undulation points during protraction before the commencement of complete retraction. Identification and analysis of Touch Induced Pumps (TIPs) was performed after low-pass filtering of whisker trajectories at 80 Hz. For quantitative analysis we chose a conservative definition of TIPs. A TIP was defined as a period within the protraction phase when both the C1 and C2 whiskers had a negative velocity. The requirement of negative velocity was adapted from [26] and [25]; here we also added the requirement of occurrence during protraction only in order to consider touch-related events and the requirement of co-occurrence in C2 and C1 in order to avoid considering events caused by mere mechanical reactions or induced by tracking noise.

Our automated analysis was verified by three independent observers who manually classified TIPs in a small subset of data (8 contact trials), after watching a short video with a few examples of TIPs. The observers were in agreement on the occurrence or absence of TIPs in 92% (515 of 557) of all touch events in the eight movies. One of the observers looked for TIPs in all the 149 contact trials, and observed a delay of 18.1±5.8 ms between touch onset and pump onset, compared to 17.9 ms with automatic detection. We considered the largest amplitude in a whisk cycle to be the onset of retraction, and so in cases where the second peak after touch was lower than the first one, this cycle was not considered as having a TIP. We noted that 27% of the TIPs that were detected manually had a second lower peak, and therefore were not detected by the automatic algorithm.

Manual detection was not limited to negative pump velocity, occurrence during protraction or co-occurrence in C2 and C1. Overall, manual classification found TIPs in 28% of the touches (compared with 18–25% by automatic detection; see Results).

### Pseudo touch control analysis

The average angle at touch onset (touch angle) was estimated for all touches in our data. For free-air whisking trials (those without an object), the time points along whisk trajectories at which whisker C2 crossed this average touch angle were identified. These crossings of the average true touch angle were later used as “pseudo-touches”, and their parameters were compared to those in real touch trials. For TIP analysis, all whisks were used (Fig. 3D, green), whereas for contact induced asymmetry analysis, only the first whisks with touch in each contact trial were used.

### Inter-TIP-interval simulated control

To validate that TIPs are clustered in time, we compared the empirical inter-TIP-interval distribution to a null model. We randomly tagged a whisk with touch as having a TIP with a probability of 25%, and then estimated the expected inter-TIP-interval statistics, by repeating this procedure 20 times (see Fig. 3E, green).

Finally, throughout the paper, mean (μ) values are presented as mean ± standard error (SE), unless otherwise noted.

## Results

To characterize the effects of touch on whisking - both within whisk and over successive whisks - we recorded the whisker movements of seven head-fixed adult Wistar rats, using a high-speed camera in the dark (see Materials and Methods). All but whiskers C1, C2, and D1 on both sides were trimmed (Fig. 1A), and the intact whiskers were tracked during whisking in air and against an object for a total of 35 minutes across 255 trials. We analyzed whisking in 106 free-air trials (Fig. 1C), and the effects of touch on whisking in 149 contact trials (Fig. 1D) in which the rats, were presented with a pole that moved towards them from the front (Fig. 1A). Whisking trajectories were filtered and individual whisks were identified automatically in the tracked data (N = 16,751 and N = 33,688 whisks in free air and contact trials, respectively).

In naturalistic whisking experiments, where rats are moving freely, single whisk trajectories vary significantly [6], [25], [28]. Fig. 2 shows examples of such whisks from freely moving rats (data taken from [6]), which do not display a simple unimodal shape, but rather exhibit multiple ‘pumps’. These pumps could result from either head or whisker movements against steady objects. Thus, whether the head or whisker is the active component generating a pump cannot be always resolved in freely moving rats.

In order to characterize the effects of touch on active vibrissal touch, void of possible confounding effects due to head-movements, we analyzed whisking of head restrained rats in the absence (free air) and presence (contact) of an object. Our study of the whisking – touch – whisking loop describes fast extra peaks induced by touch, with kinematics that is much faster than that of the doubled-peaked whisks observed in freely-moving rats [25]. The whisking angle was filtered at 15 Hz for delineation of whisks. For the detailed analysis of within cycle modulations we used 80 Hz filters (see Methods).

### Touch Induced Pumps (TIPs): Fast feedback after whisker contact in the ipsilateral side

After touching the pole for the first time, our rats typically palpated against it for several whisk cycles with repeated touch-detach events. The mean number of uninterrupted (but with detach), sequential whisks-with-touch was 5.5 cycles (N = 7 rats, range 2.8–9.6 cycles). In some cases, the sign of whisking velocity changed for a brief period (typically <10 ms) after touching an object (Fig. 3A–C and Movie S1). This short period of negative velocity was followed by a period of positive velocity, until the maximal angle of the whisk was reached - resulting in an extra peak or “second pump” within the protraction phase. We named such an event as a Touch Induced Pump (TIP). In all these cases the whisker stayed in contact with the pole throughout a second pump. Detection of TIPs was done automatically from the trajectory of the whisker angle using well-defined criteria as well as by human observers, examining the movies (see Materials and Methods). Unless stated otherwise, the TIPs presented here are those passing the automatic criteria. A peri-contact time histogram shows there was an abrupt and significant increase in the probability of a TIP after the onset of contact (mean delay 17.9 ms; Fig. 3D). The delay of manually detected TIPs, from touch onset to TIP onset, was 18±5.8 ms (80% were in the range of 14–22 ms; see Fig. S1). Visual inspection of whisks with and without TIPs (e.g., 3B) cannot resolve whether TIPs were initiated during protraction or retraction. We thus compared whisker velocities during the initiation of TIPs and retractions in whisks with touch but no TIPs. The velocity of the negative slope of TIPs was significantly smaller than that of retractions onset (162 deg/s versus 595 deg/s for non-contacting whisker C1; p<10^−6^, Wilcoxon rank sum test). This indicates that the negative velocity in the pump is unlikely to be the beginning of the “end-of-whisk” retraction.

TIPs occurred in 25% of whisks with touch (Fig. 3D, magenta bars).In order to estimate the probability that a TIP will occur by chance, we identified moments during free-air trials as “pseudo-touch” moments (see Materials and Methods). In 7% of these free-air “pseudo-touch” whisks a “pseudo-TIP” was detected (Fig. 3D, green bars). We conclude that significant proportion of post-contact pumps was induced by contact itself. The observed probability of TIPs (25%) is, however, likely to be an underestimate of the fraction of TIPs induced by object contact. First, only TIPs in which a simultaneous negative velocity within protraction in whiskers C2 and C1 (although C1 rarely touched the object) occurred were considered. Second, double pumps in which the first post-touch peak was higher than the second post-touch peak were omitted, since we only considered double pumps that occurred before the maximal protraction point. In 60% of the cases in which C1 displayed a TIP, a TIP was identified in D1 as well. In only 8% of C1 TIPs the contralateral whiskers displayed a TIP. In general, contralateral TIPs occurred at a rate not significantly different than that predicted by pseudo contacts (8% Vs 7%).

### Temporal clustering of TIP

TIPs were often clustered in time (Movie S1), as shown by the typical inter-TIP interval being significantly shorter than predicted from random distribution (Fig. 3E). Looking at the manually detected TIPs we further noted that the probability that a whisk had a TIP was higher if any of the recent whisks had TIPs: If the most recent whisk, but not one before it, had a TIP, the probability of a TIP in the current whisk was 42%. If the last two whisks had a TIP, then the probability for a TIP in the current whisk was 58%. For a series of whisks with touch, the probability for a TIP was 20% in early touches and 34% in late ones (early and late were defined here as before and after the median number of touch in a contacting bout). We note that while the pole was moving only in early touches, the probability of a pump was significantly higher in late touches, which rules out the possibility that pole motion directly enhances pumps.

### Whisks with TIPs contact longer and begin at more protracted set points

**Figure 4:**
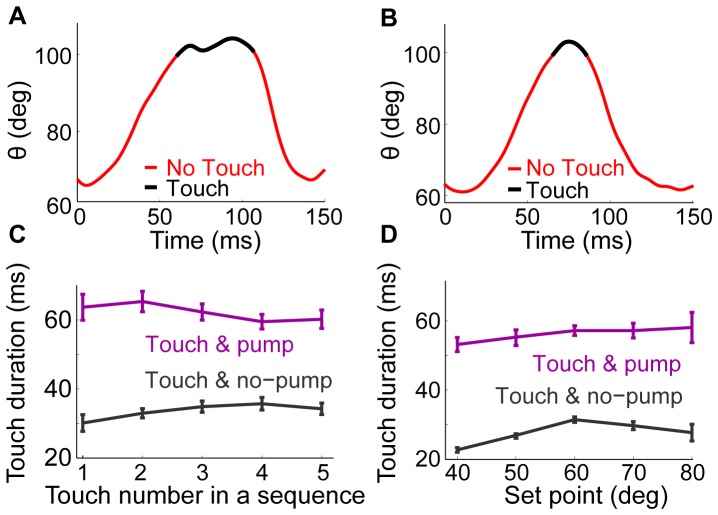
Contact durations are longer in whisks with TIP. **A.** A typical single whisk with a pump after touch onset. Period of whisker-pole contact marked in black. The pump occurred while the whisker stayed in contact with the pole. **B.** A typical single whisk without a pump after touch onset. **C.** Touch duration as a function of the position of the touch in a sequence of touches. Whisks with touch and TIP (purple) and whisks with touch but no TIP (gray). **D.** Touch duration with and without a TIP, as a function of the set point.

**Figure 5:**
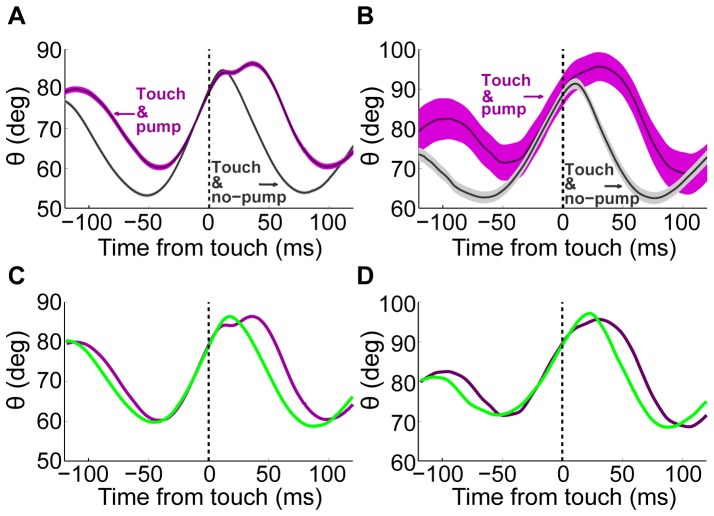
TIPs occur more often in whisk cycles with higher set-points. **A.** The average angle trace of whisker C1 (not touching) triggered on the onset of touch by whisker C2 for all whisks with (purple; mean ± SE) and without pumps (grey; mean ± SE). **B.** Similar to A, but when only data from the first touches in a trial (N = 140) were used. **C.** Mean trace around touch with TIP (purple) and over a subset of traces around touch with no TIP (control; green) for the data in A. The control subset was chosen by removing traces with the lowest set points, so that the mean set points of the control (green) and pump (purple) traces will be equal. **D.** Similar to C, but when only data from the first touch occurring in a trial was used.

In all whisk cycles with a TIP, the whisker stayed in contact with the pole throughout the TIP, and only detached from the pole during retraction. Thus, a TIP contained a single contact epoch in which the contact pressure was modulated (see Figs. 3C, 4A, and Movie S1). The total duration of pole contact was twice as long for touches with TIPs (55.5±1 ms) than for touches without TIPs (27±0.4 ms; Figs. 4A–D; see also average traces in Figs. 5A–B). The increase in touch duration was significant for all touches in a bout (Fig. 4C; p<0.001, two-sample t-test) and was weakly affected by the set point (Fig. 4D; p<0.001, two-sample t-test).

We found a higher probability for a TIP in whisks that had a more protracted angle at the beginning of the protraction, or “set-point” (Fig. 5A, see also methods). The average protraction set point in whisks with a TIP was 7.3 deg higher than that of TIP-less whisks. These more protracted set points for whisks that had a TIP are evident already at the first touch (Fig. 5B). Thus, the more protracted set point cannot be explained in terms of the effect of previous whisks with TIPs.

The mean time between protraction onset and touch onset for whisks with TIP was 36.1±15.4 (mean ± SD) ans 49.5±18.6 ms for TIP-less ones. The occurrence of TIPs was not correlated with the angle at touch. To find the net effect of pump on whisking after touch, we compared the average trace of whisks with pump to that of the control whisks with pseudo-touch that start at high set-point (Figs. 5C–D). The duration of protraction and that of the whole whisk were longer for whisks with TIPs than for whisks with touch, but no TIP. Protractions lasted 89.1±0.8 ms versus 76.2±0.4 ms, and the whisks of the non-contacting whisker C1 were 150.3±1.2 ms long versus 142.4±0.6 ms (p<0.01, two sample t-test). The amplitude was slightly smaller for whisks with a TIP than those without (31.1±0.7 versus 34.4±0.3 deg; p<0.01, two sample t-test; Fig. 5A). This amplitude difference was probably due to the more protracted set point, rather than due to the TIP. The correlation of the set point and amplitude was ρ = 0.63 (p<0.01) while no significant effect was found for the existence of TIP on the amplitude when whisks with similar set-points were compared (30.3±0.3 deg vs. 31.1±0.7 deg, p = 0.22; Fig. 5C).

### Temporal and spatial correlations during free-air whisking

**Figure 6:**
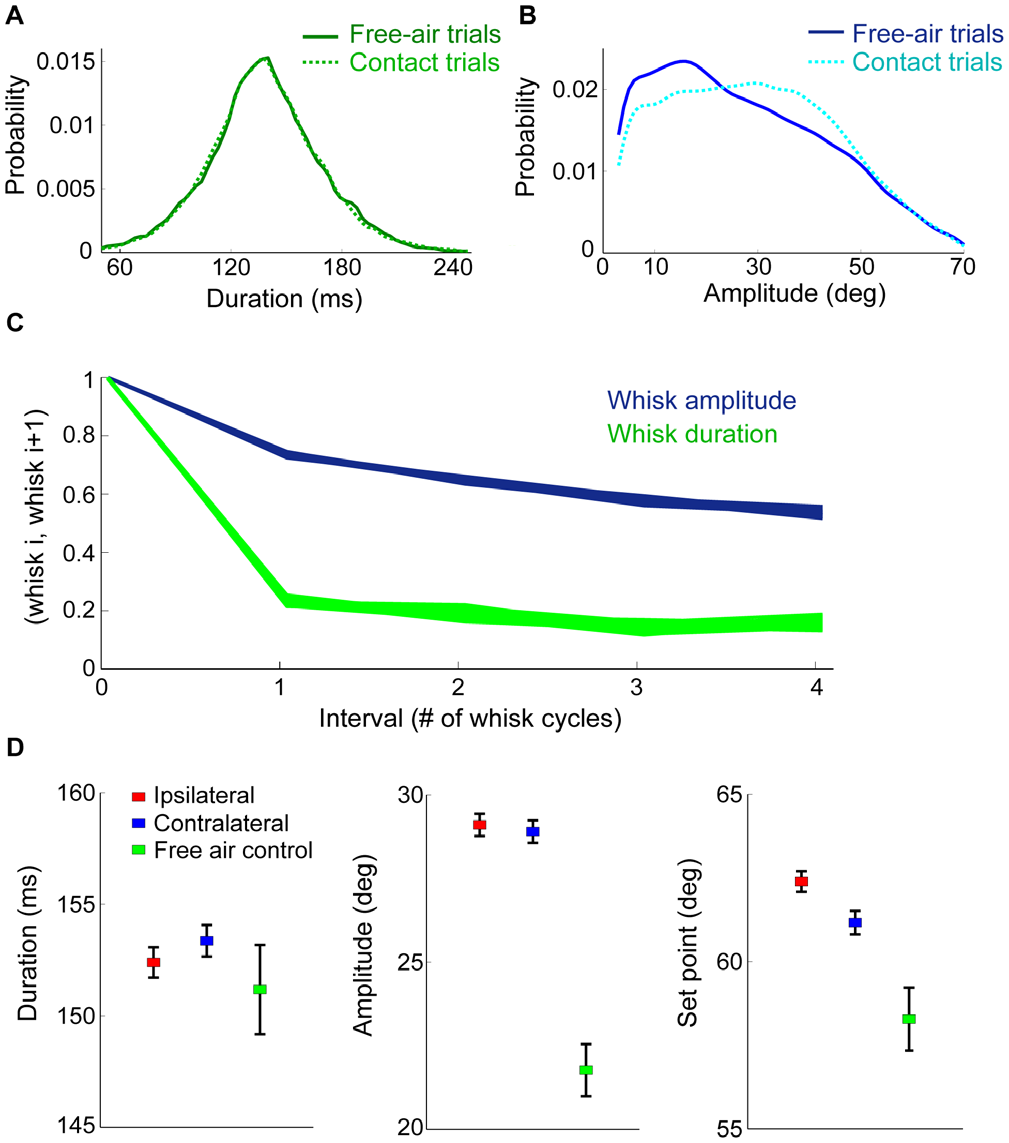
Whisking kinematics in free-air and after contact. **A.** Distribution of whisk duration in free-air (dark green) and in contact (light green) trials. **B.** Distribution of whisk amplitudes in free-air (dark blue) and contact (light blue) trials. The distributions in A and B where smoothed using the MATLAB function *fitdist* (‘kernel’, width = 2). **C.** Correlations between the amplitude and duration of pairs of whisks, as a function of the interval between them. **D.** Whisk duration, amplitude, and set point, before the first “pseudo touch” (free-air trials; green) and the first (real) touch (contact trials; red for the ipsilateral and blue for contralateral sides).

In addition to the TIPs, we explored the effect of touch on the whisking patterns over successive whisks, and across sides. First we studied free-air trials, in which whisk duration was nearly normally distributed (148.7±30.2 ms; mean ± SD), with a long tail at large duration values and an average CV = 0.2 (CV – coefficient of variance; ranging from 0.17 to 0.23 for individual rats; Fig. 6A). The mean protraction and retraction durations were 78.4±18.1 ms and 70.3±21 ms respectively. However, the protraction amplitude had a wide range of values (28.4±16.4 deg, and its distribution was clearly non-Gaussian (Fig. 6B), and a CV of 0.58 (ranging from 0.4 to 0.63 for individual rats). For all animals, whisking amplitudes were highly correlated with the maximal whisk velocity for both protraction (ρ = 0.92, where ρ is the correlation coefficient) and retraction (ρ = 0.96), see also [13]) and [29]). In contrast, the correlation between whisk amplitude and duration was low for both protraction (ρ = 0.2) and retraction (ρ = 0.02). The correlation between protraction duration and retraction duration was also low (ρ = 0.17), while the amplitudes of protraction correlated highly with those of retraction (ρ = 0.91). Moreover, while whisk amplitude was higher in whisks from contact trials, the whole whisk duration was not affected by a touch (Fig. 6A–B). These findings are consistent with previous suggestions that the control of whisking amplitude and whisking velocity are strongly coupled, and are independent from the control of whisking duration [22], [30].

During free-air whisking, the amplitudes of successive whisks were highly correlated (ρ = 0.74), and whisk durations only weakly so (ρ = 0.23). These whisk correlations decayed with the temporal separation between whisks (Fig. 6C). Linear predictive models of whisking amplitude, based on previous whisks, were slightly better when they used the last five whisks, compared to models that used just the most recent whisk (data not shown).

We found that whiskers on the same side were more synchronized with each other (ρ>0.9), than with the corresponding whiskers on the opposite side (ρ<0.6, N = 97 pairs), as previously reported [15]. An example of the trajectory of four whiskers (three on the same side and one on the opposite side) is depicted in Figure S2A. Neighboring whiskers in the same row (C1–C2) were significantly more correlated than whiskers in the same arc (ρ = 0.98±0.002, N = 205 C1–C2 pairs, compared to ρ = 0.93±0.007, N = 132 C1–D1 pairs, p<0.001, Wilcoxon two-sided rank sum test; see also Fig. S2C and [31]. This finding seems consistent with the organization of whisker musculature, whereby neighboring whiskers are more strongly inter-connected by the intrinsic musculature along rows than along arcs [32], [33], [34]. The correlation between the amplitude of whisks was higher for whiskers on the same side, compared to whiskers on opposite sides (Fig. S2D).

### Slow, bilateral effects of touch

**Figure 7:**
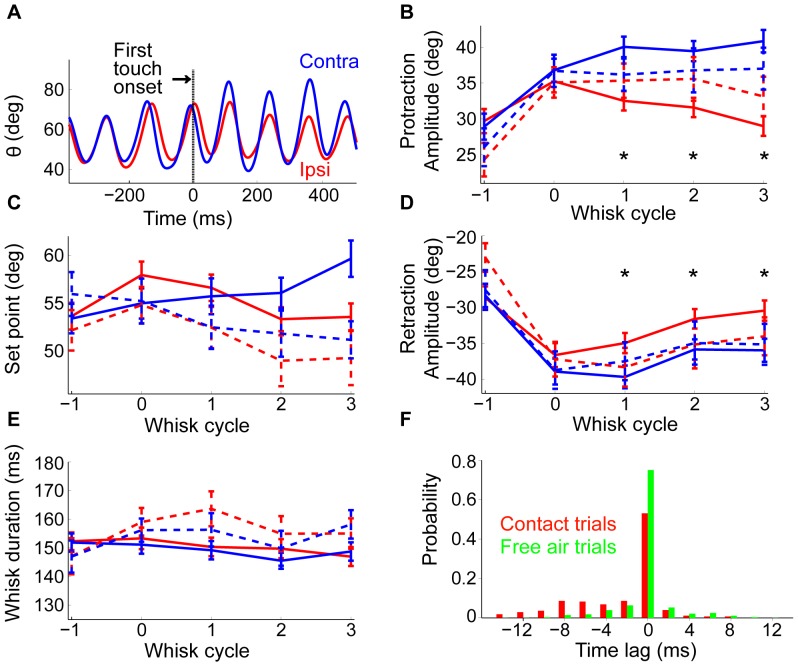
Whisking amplitudes, durations, and set-points after first touch. **A.** Whisking angle of the ipsilateral C1 whisker (red) and contralateral C1 whisker (blue). Onset of the first touch in a trial is marked by a vertical line. **B.** Protraction amplitudes of whisker C1 as a function of whisk cycle, around first touch (red, blue) or first pseudo touch (dotted red, dotted blue). Ipsilateral C1 whisker (red, dotted red) and contralateral C1 whisker (blue, dotted blue). The most rostral untrimmed whisker C2 is touching the pole (the rare trials where whisker C1 also touched the pole in any of the first contacts were excluded from this analysis). Whisk cycle 0 is the first whisk with touch (or pseudo touch) in the trial. Asterisks indicate significant difference between ipsi and contralateral sides. We note that since the number of pseudo-touches was smaller than the number of real contacts, the error bars in the pseudo-touch trials were usually larger. **C.** Set point around touch as a function of whisk cycle. **D.** Same as B, but for retraction amplitude (negative value indicates retraction). **E.** Whisk duration around first touch. **F.** Probability of time-lags for contact and free-air trials.

Next, we examined the simultaneous whisking patterns on the ipsilateral and contralateral sides, before and after the first whisk with a touch in a trial. Our rats increased whisking set points and amplitudes upon the initiation of pole movement, an event that was perceivable to the rat via the associated acoustic noise (Movie S2). This effect is demonstrated by comparing whisking parameters between contact trials and free-air trials, before the first contact (or pseudo-contact). The comparison revealed significant increase of set point and amplitude but not duration (Fig. 6D). Furthermore, whisking was typically symmetric between sides before the first contact. After the first contact, whisking amplitudes increased on the contralateral side and decreased on the ipsilateral side across successive whisks, consistent with [16]. We quantified this contact induced asymmetry by measuring the protraction and retraction amplitudes of the whisks. To rule out the possibility of direct mechanical effects of touch on the ipsilateral contacting whisker (C2) we compared the amplitudes of the non-contacting whisker C1 on both sides (omitting trials in which C1 touched the pole in the first contacts). We found that protraction amplitude increased on the contralateral side (Figs. 7A–B and Movie S2), starting at the first whisk cycle after the cycle with touch (cycle “1”). In contrast, on the ipsilateral side the protraction amplitude decreased starting at cycle 1. The difference between the amplitudes on the ipsi- and contra-lateral sides (mean 9.2 deg) was significant for every cycle (1–3; p<0.01, paired t-test for each cycle), whereas the differences between the amplitude in each side and its pseudo-touch control (Fig. 7B dashed curves) were significant only for the data pulled over the 3 cycles (p<0.05, unpaired t-test). This finding is consistent with previous results, measured in freely moving rodents [16], [35]. The difference in set point between contact and free-air trials was not affected by contact (Fig. 7C, compare to Fig. 6D). The behavior of retraction amplitudes mirrored that of protraction amplitudes (Fig. 7D). Interestingly, whisk duration remained coordinated between the ipsilateral (145.1±2.1 ms) and contralateral (146.4±2 ms) sides during the three whisks following the first touch cycle (p = 0.42, paired t-test; Fig. 7E).

Finally, we analyzed the effect of contact on bilateral synchrony by measuring the difference in protraction onsets, or time lag, between sides. Time lags were calculated for all pairs of simultaneous ipsilateral and contralateral whisks that had a temporal overlap of more than 80% (the result did not significantly change in the range 50–95%). The difference between the time lags in contact trials, after the first contact (mean = 2.22 ms, median = 0), and the time-lags in free-air trials (mean = 0.03 ms, median = 0) was small, but significant (P<0.01, Wilcoxon rank sum test, Fig. 7F). In most cases the lag was negligible, but in a few cases the contralateral side lagged behind the ipsilateral following the first touch (negative values indicating lagging of the contra-lateral side).

## Discussion

High-speed videography has revealed that whisking is more complex and adaptive than previously assumed [16], [25], [28]. Here, we examined the effects of active touch on whisking in head-fixed rats by comparing free-air whisking prior to contact with whisking patterns following contact. We found that actively touching an object caused changes in whisking patterns on both the ipsilateral and contralateral sides, at two different timescales. On the ipsilateral side, active touch frequently resulted in an additional synchronous deflection pump of all tracked whiskers ∼18 ms after contact. These touch-induced pumps (TIPs) prolonged the total duration of contact significantly, were exclusively ipsilateral, although not restricted to the contacting whisker. It should be emphasized that these TIPs differ qualitatively from the ‘double-pumps’ reported in free-air whisking rats [25]. While TIPs are triggered by touch, free-air ‘double-pumps’ are not. On a longer timescale, whisking amplitudes increased on the contralateral side and decreased on the ipsilateral side. This contact induced asymmetry in whisking amplitude is consistent with previous results in freely-moving rats [16], and might be related to the ‘look ahead’ behavior reported by [15] in freely exploring rats. Both the ipsilateral TIPs and the contact induced asymmetry can be considered as components of active touch, whereby the sampling of the world changes following an external sensory input, in this case both within and across whisking cycles.

The sensory-motor vibrissal control system encompasses multiple closed loops that connect the sensory periphery to motoneurons through different levels in the nervous system [36]. Inevitably, all these loops participate to some degree in the active control of whisker motion. The effects of sensory inputs on whisker motion can therefore be expressed after various delays, and extend across various time scales, depending on loop length and ongoing activity. The specific types of active whisking feedback we describe here vary both in time and space. A fast, within-cycle, ipsilateral whisker pumping occurred in a significant proportion of whisk cycles when contact occurred (Figs. 3, 4, and 5). A slower, across-cycle, bilateral modulation of whisk amplitudes occurred after first contact (Fig. 7). The different delays, time scales, and spatial characteristics of these effects suggest that they are mediated through different sensory-motor loops of the vibrissal system.

### Possible neural pathways of TIPs

In principle, TIPs could be the result of mechanical effects alone, such as whisker resonance [37], [38]. We rule out mechanical effects, however, since TIPs only occurred in a subset of whisk cycles, and were temporally clustered (see below). Moreover, similar TIPs were observed simultaneously in the C2 whisker (touching) and in C1 (non-touching), and in *at least* 60% of the cases also in D1 (non-touching different row). In fact, the average pump amplitude was slightly larger for C1 (1.5 deg) than for C2 (1.2 deg) and the latencies were comparable (see Fig. 3C). The possibility that the pumping of C1 followed that of C2 via tissue coupling was ruled out by analyzing experiments with artificial whisking, where stimulations of facial nerves moved the whiskers against objects [39]. Blocking a single whisker (close to its origin) had a negligible effect on neighboring whiskers (Fig. S3). These observations suggest a neural source for touch-induced pumps.

Given the short delays of TIPs (∼18 ms), they are likely to be mediated by a neural loop that is closed at the level of the brainstem [24],[40]. The brainstem loop is the shortest anatomical pathway between the mechanoreceptors and muscles associated with the whiskers, and its temporal path-length matches that of TIP delays, making it a likely candidate for mediating these fast modulations. The shortest brainstem loop would encompass the mechanoreceptors of the whisker follicles, primary afferents with cell bodies in the trigeminal ganglion (TG), projections neurons of the trigeminal nuclei (TN), motoneurons of the facial nucleus (FN) and the mystacial muscles (MM) [36]. In young rats (P7 to P15), sensory signals traverse this loop (mechanotransduction excluded) at latencies between 5 and 15 ms [24]. We found that the time from touch to the first peak in 80% of the TIPs was 14–22 ms, while significant differences in movement trajectories between whisks with and without TIPs were seen as early as 10 ms after touch onset (Fig. 5C). Furthermore, the fast, within-cycle feedback was exclusively ipsilateral, which is consistent with projections from the TN to FN being almost exclusively ipsilateral [41].

Alternatively, TIPs may be mediated by longer loops. Superior colliculus (SC) neurons respond to passive whisker stimulation with delays as short as 2–8 ms [42], and the neural latencies from the SC to the FN can be as short as 5 ms [31]. Estimating the delay from FN spiking to actual whisker movement at ∼5 ms [43] and adding to that intra-SC delays suggests that a loop through the SC could also mediate TIPs with latencies as short as 13–19 ms, consistent with our behavioral data.

In contrast, a loop via the cortex is unlikely to mediate TIPs. The sum of the shortest delays from whisker stimulation to cortical activity (∼10–15 ms; [44], [45]) and from cortical stimulation to whisker movement (>25 ms; [46]) exceeds, by far, the fast feedback we observe here.

### Possible neural mechanisms of TIPs

Since TIPs typically occurred near the peak of a whisking cycle, they could be interpreted as either a forward movement that occurred after the whisker started to retract, or a backward movement that started before the end-of-whisk retraction. However, the negative slope after the first peak was four times smaller than the slope at the beginning of the end-of-whisk retraction (see *Touch Induced Pumps (TIPs): Fast feedback after whisker contact in the ipsilateral side* in Results), suggesting that TIPs start during the protraction phase. TIPs could be miniature ‘extra-protractions’ (Fig. S4A) or miniature ‘extra-retractions’ (Fig. S4B) modulating the main protraction cycle, shortly following touch. These miniature events could be mediated by a small number of additional spikes generated by the FN motoneurons driving intrinsic or extrinsic muscles [24], [43].

Whisking results from triphasic activation of mystacial musculature [47]. Upon whisker protraction, the rostral extrinsic *m. nasalis* muscle pulls the whisker pad forward, followed by a contraction of intrinsic muscles that pivot the whiskers further rostral. The third phase involves relaxation of both extrinsic and intrinsic protractors and contraction of the *m. nasolabialis* and *m. maxillolabialis* muscles, which together pull the whisker pad back. In the context of such triphasic muscle recruitment, TIPs can be restated in terms of muscle activation. First, a TIP could result if there was a temporal discontinuity between the recruitment of the extrinsic and intrinsic protractors. This would result in two discrete, non-overlapping phases of whisker protraction. This scheme assumes that a contact that occurs during the initial protraction phase delays the onset of the second (intrinsic) protraction phase via inhibitory connections affecting the CPG. Alternatively, the later part of extrinsic protraction or early part of intrinsic protraction could be suppressed by direct inhibition of FN motoneurons by inhibitory TN projection neurons [40]. A TIP could also be generated by direct, phasic excitation of protractor or retractor motor or pre-motor neurons. In the case of excitation of protractor neurons, this would produce a second accelerative event during the first and/or second phase of a whisk. In the case of excitation of retractor neurons, the effect would be an interruption of the ongoing whisker protraction, and possibly a reversal of movement direction.

Excitation of protractor neurons, or inhibition of retractor neurons, would mimic a behaviorally-observed TIP only if occurring early in protraction (Fig. S4A). Excitation of retractor neurons or inhibition of protractor neurons, in contrast, would mimic behaviorally-observed TIPs regardless of their timing during the protraction phase (Fig. S4B). In our data, TIPs almost always occurred in the second half of protraction, lending support to inhibition of protractors or excitation of retractors. Although anatomical studies suggest that both excitatory and inhibitory feedback can occur through a TG-FN loop, only excitatory feedback has been functionally demonstrated *in vivo*, notably onto both protractor and retractor neurons [24]. A more recent study has confirmed that TN pre-motor neurons in SpVi exert an excitatory effect on retractor neurons in the awake, behaving mouse [48]. Thus, although none of the mechanisms suggested above can be ultimately ruled out at this stage, two schemes stand out as more plausible given existing data: TIPs are mediated via excitatory feedback onto retractor motor or pre-motor neurons and/or via inhibitory feedback onto the CPG delaying the retraction phase. The observation that in whisks with TIPs both transient retraction and a delay of the full retraction typically occur (Fig. 5) suggests that both feedback circuits are activated in these cases.

### Top-down control of TIPs

Two observations suggest that the execution of TIPs might be controlled by higher circuits: TIPs were temporally clustered (Fig. 3E) and associated with a protracted set-point (Fig. 5). Furthermore, the higher probability of pumps at later touches may indicate ‘switching’ between two modes, one that blocks TIPs and one that enables them. Such gating could be mediated by descending cortico-trigeminal projections [49], [50], [51], [52], [53] or cholinergic inputs to the TN from the pedunculo-pontine nucleus [54]. Such projections can mediate both the protracted set-point and the enabling of TIPs by simply exciting neurons in the trigeminal nuclei which project to the FN and/or the CPG [23]. It should further be assumed that the projections of the sensory channels carrying touch information are selective, in the fashion suggested in the previous section.

### Neural mechanisms of contact induced asymmetry

An increase in whisking amplitude was observed in the contralateral side following contact, while a small decrease in the amplitude was observed in the ipsilateral side. This effect on the ipsilateral side might be, at least in part and for the first touches, a reflection of predictive whisking trying to follow the trajectory of the moving pole.

Unlike the fast feedback of TIPs, the slower, across-cycles, and bilateral changes in whisk amplitudes following contact is likely to involve loops with longer temporal path-lengths and slower dynamics, including those circuits that possess mechanism capable of retaining sensory events across multiple whisk cycles [22], [55], [56], [57], [58]. On the other hand, elaborated cortical processing may not be required to express these behaviors. In a recent study, Mitchinson et al. [35] found that asymmetric whisking during head turning and following unilateral object contacts are present also in the grey short-tailed opossum, whose brain has a smaller number of cortical areas than most mammals, lacks a distinct motor cortex, and has relatively few corticospinal neurons.

### Functional role of fast feedback

The fast whisking exhibited by rodents provides a mechanism to quickly scan the immediate environment for behaviorally relevant features, be it for avoiding obstacles during locomotion [16], detecting prey/food [59] or interacting with peers [60]. Ballistic, rhythmic whisking may not be optimal, however, in all sensory tasks. Object identification, for instance, may require persistent contact with objects. Here, we observed rats intermittently exhibit an intermediate behavior between ballistic periodic whisking and persistent contact. In about quarter of the contacting whisks, typically in those occurring late into epochs of periodic whisking, small pumps were observed. These pumps led to a doubling (addition of 103%, or 28 ms on average) of the period each whisk was in contact with the object, while at the same time increasing whisk duration by only 5% (or 8 ms on average). In our paradigm initial contacts with the object were usually brief (without TIPs), obeying a ‘minimal impingement’ principle [16], while prolonged exploration often involved longer contacts (via TIPs), which can facilitate object characterization (see also [9]). The dependencies of these dynamics on object familiarity [61], identity or context are yet to be determined.

## Supporting Information

Figure S1 **Manual detection of TIPs.** Distribution of latencies from touch onset to pump onset in contact trials, as found by visual inspection of the slowed-down (×10) video files. The average latency of TIPs over all manually detected pumps was 18.1 ms.
(EPS)

Figure S2 **Correlations of neighboring whiskers during free-air whisking.** **A.** Joint angle trajectory, from a free-air trial, of four whiskers during 2.1 seconds. Three whiskers (C2 in red, C1 in black, D1 in green) were on the left side of the snout (ipsilateral) and one (C2 in blue) was on the right side (contralateral). **B.** Section of a mystacial pad (blood sinuses that surround whisker roots where visualized using Xylene; Courtesy of S. Haidarliu, Weizmann Institute of Science). Colored dots indicate the locations of whiskers C2 (red), C1 (black) and D1 (green). **C.** Correlation between angle trajectories of whisker pairs, on the same and opposite sides of the snout. Thin black lines indicate standard error values, which were estimated over all trials **D.** Correlations between protraction amplitudes (rather than entire angle trajectories) for the same pairs of whiskers as in C. For a given pair of whiskers, amplitudes within pairs of same (simultaneous) whisks (overlap in time >80%) was compared.
(EPS)

Figure S3 **Weak mechanical coupling between adjacent whiskers.** Blocking of a single whisker has no significant effect on the movement of a neighboring whisker. In this experiment (Szwed M., Bagdasarian K., Ahissar, E, unpublished), an anesthetized rat was artificially whisking (as a result of facial nerve stimulation; see Szwed et al., 2003). Blocking whisker C1 by inserting a pole close to the whisker base (black), did not affect the movement of C2 (solid red; compare to C2 whisking when C1 is not blocked, in dashed red).
(EPS)

Figure S4 **Models of the functional mechanism underlying TIPs.** **A.** Potential source of TIPs as a positive feedback mechanism is shown by a simulated whisking trajectory, in which miniature half-cycle cosine whisks (black; amplitude of zero between the half cosines) were added at different phases of protraction phase in the whisks (summed trace shown in purple). **B.** Potential source of TIPs as a negative feedback mechanism is shown by a simulated whisking trajectory, in which miniature half-cycle cosine whisks a (black; amplitude of zero between the half cosines) were subtracted at different phases of protraction phase in the whisks (summed trace shown in purple). For reference, a free-air whisking trace (green) that contains three whisks is shown in both panels.
(EPS)

Movie S1 **Example of whisking containing several TIPs in the second half of a trial.** The rat is palpating against the pole. This 2 s long movie was taken at 1000 frames per second (fps), at a resolution of 480×640 pixels and then compressed and slowed down to 12 fps. For clarity, only 4 whiskers where tracked in this example, and the corresponding angle traces are shown below (ipsilateral C2, C1 in blue and green, contralateral C2, C1 in cyan and purple. TIPs are can be seen in the movie and the corresponding traces in 12 out of the 13 whisks.
(MP4)

Movie S2 **Example of contact induced asymmetry.** The movie is from the first part of a single trial. The amplitude of whisking increased on both sides shortly after the pole started to move (perceivable by the rat because of the motor's noise), and increased further on the contralateral side after the first touch. The asymmetry in amplitude can be clearly seen in the movie. The first touch in the movie occurs after ∼1 sec. The movie shows 3 sec of whisking, sampled at 500 fps. Resolution and video compression are as in Movie 1; movie was slowed down to 25 fps.
(MP4)
